# Neoadjuvant chemotherapy followed by concurrent chemoradiotherapy versus concurrent chemoradiotherapy alone in nasopharyngeal carcinoma patients with cervical nodal necrosis

**DOI:** 10.1038/srep42624

**Published:** 2017-02-17

**Authors:** Mei Lan, Chunyan Chen, Ying Huang, Li Tian, Zhijun Duan, Fei Han, Junfang Liao, Meiling Deng, Terence T. Sio, Anussara Prayongrat, Lie Zheng, Shaoxiong Wu, Taixiang Lu

**Affiliations:** 1 State Key Laboratory of Oncology in Southern China, Sun Yat-sen University Cancer Center, Collaborative Innovation Center for Cancer Medicine, Department of Radiation Oncology, Guangzhou, China; 2 State Key Laboratory of Oncology in Southern China, Sun Yat-sen University Cancer Center, Collaborative Innovation Center for Cancer Medicine, Imaging Diagnosis and Interventional Center, Guangzhou, China; 3 Chengdu Military General Hospital, Department of Radiation Diagnosis and Interventional Center, Chengdu, China; 4 Mayo Clinic, Department of Radiation Oncology, Scottsdale, Arizona; 5 Division of Radiation Oncology, Department of Radiology, Faculty of Medicine, Chulalongkorn University, King Chulalongkorn Memorial Hospital, King Bangkok, Thailand

## Abstract

The effectiveness of neoadjuvant chemotherapy (NACT) followed by concurrent chemoradiotherapy (CCRT) compared with CCRT alone in nasopharyngeal carcinoma (NPC) patients who presented with cervical nodal necrosis (CNN) is unknown. A total of 792 patients with stage T1-4N1-3M0 NPC and presented with CNN based on magnetic resonance imaging were retrospectively reviewed. Propensity score matching method was used to balance treatment arms for baseline characteristics. Eventually, 508 patients were propensity-matched on a 1:1 basis to create two groups (NACT + CCRT and CCRT groups). Survival rates were calculated by Kaplan–Meier method and differences were compared by using the log-rank test. The 5-year disease specific survival, disease-free survival and distant metastasis-free survival were significantly higher in NACT + CCRT group relative to the matched CCRT group (82.1% vs. 72.5%, P = 0.021; 70.3% vs. 54.1%, P < 0.001; 81.9% vs. 67.3%, P < 0.001, respectively). Although the rates of grade 3–4 leucopenia and mucositis were higher in NACT + CCRT group than CCRT group, compliance with the combined treatment was good and no significant difference was observed between two groups. NACT followed by CCRT was relatively safe and could achieve better survival than CCRT alone in NPC patients with CNN by reducing the risk of death, tumor progression and distant metastasis.

Due to the particular anatomical location and symptoms of nasopharyngeal carcinoma (NPC), most patients with NPC typically have loco-regionally advanced disease at diagnosis^1^. Approximately 75–85% of NPC patients have regional lymph node metastases^2^,^3^, and up to 44% of these patients reported concurrent cervical nodal necrosis (CNN)^4^. CNN has previously been reported as a strong, independent negative prognostic factor for overall survival (OS), disease free survival (DFS) and distant metastasis-free survival (DMFS). CNN patients have previously been significantly associated with an approximately 12% reduction in 5-year OS, DFS and DMFS rates relative to non-CNN patients^4^. Furthermore, approximately 20% of CNN patients subsequently develop metastatic disease^4^. Given the poor prognosis, it is important to find effective strategies to improve the survival outcomes of this specific subgroup.

Concurrent chemoradiotherapy (CCRT) has been settled as a standard treatment and recommended as a category 2A option for locally advanced NPC^5^. The five-year OS rate under CCRT could be increased from 59% to 70% when compared to radiation (RT) alone^6^,^7^. However, although adding neoadjuvant chemotherapy (NACT) is considered to have the potential to further reduce the risk of distant failure, especially in patients with extensive nodal disease, the value of NACT followed by CCRT remains controversial^8^,^9^,^10^,^11^. Some studies have reported that NACT may significantly decrease the risk of distant metastasis in locally advanced NPC in addition to CCRT^8^,^12^,^13^, but others failed to show any survival benefit^14^,^15^.

Since the value of NACT in NPC patients with CNN has not been clearly demonstrated, we performed this retrospective study to evaluate the role of NACT followed by CCRT in this specific group of patients.

## Results

### Treatment outcomes

Median follow-up time for the 508 propensity score-matched patients was 50 months (range, 4.5–86 months). At last follow-up, 183 patients (36.0%, 183/508) had experienced treatment failure and 111 (21.9%, 111/508) had died. A total of 74 patients (14.6%, 74/508) developed loco-regional recurrence and 127 patients (25.0%, 127/508) developed distant metastases. The patterns of treatment failure were summarized in Table 1. Fewer patients (*n* = 71) in NACT + CCRT group experienced treatment failure compared with CCRT group (*n* = 112), with a lower rate of distant metastases (19.3% vs. 30.7%, *P* = 0.004). But no difference was found in loco-regional recurrence rate (*P* = 0.530).

**Figure 1:**
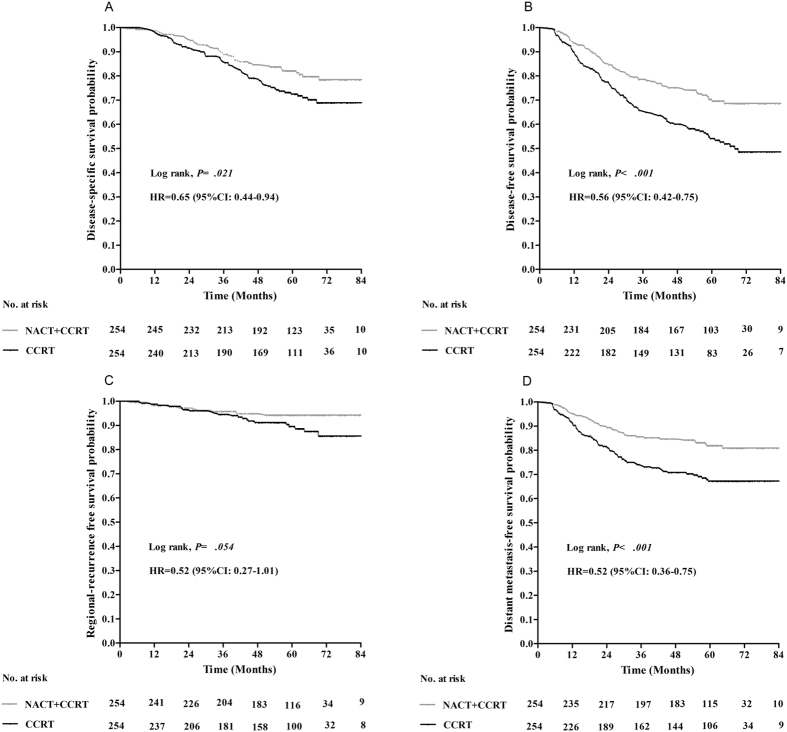
Kaplan–Meier disease-specific survival (**A**), disease-free survival (**B**), regional recurrence-free survival (**C**) and distant metastasis-free survival (**D**) curves for the NACT + CCRT group and CCRT group.

For the entire matched cohort, the 5-year DSS, DFS, RRFS and DMFS rates were 77.4%, 62.3%, 92.1% and 74.7%, respectively. The survival rates of 5-year DSS, DFS and DMFS were significantly higher in NACT + CCRT group relative to the CCRT group (82.1% vs. 72.5%, *P* = 0.021; 70.3% vs. 54.1%, *P* < 0.001; 81.9% vs. 67.3%, *P* < 0.001, respectively), Although no significant difference was observed in RRFS between two groups, there was an improvement tendency in regional control following NACT + CCRT compared with CCRT alone (94.3% vs. 89.6%, *P* = 0.054)(Fig. 1).

### Subgroup analysis

Subgroup analyses demonstrated that patients with different NACT regimen combinations (TPF, PF or TP) were associated with similar survival rates (Supplementary Figure 1). No difference in DSS, DFS, DMFS was observed between different NACT cycles (≤2 cycles vs. >2 cycles), with 5-year DSS, DFS and DMFS rates of 81.3% vs. 85.5% (*P* = 0.698), 71.0% vs. 68.0% (*P* = 0.698), 81.8% vs. 82.7% (*P* = 0.824), respectively. However, the 5-year RRFS was significantly higher in patients ≤2 cycles than those >2 cycles (96.1% vs. 87.3%, *P* = 0.017). (Supplementary Figure 2)

### Univariate and Multivariate analysis

Univariate analysis revealed age (>44 years), gender (male), T stage, clinical stage, treatment of NACT + CCRT were prognostic factors (Supplementary Table). Multivariate analyses demonstrated that the use of NACT + CCRT could significantly reduce the risk of death, tumor progression and distant metastasis compared with CCRT alone. The risk of distant metastasis could be reduced over 50% (HR = 0.49; 95% CI, 0.34–0.71) by addition of NACT to CCRT. T stage was an independent prognostic factor for DFS and DMFS (*P* = 0.013 and *P* = 0.003, respectively). Gender (male) was an independent negative prognostic factor for DSS, DFS and DMFS (*P* = 0.01, 0.028 and 0.008, respectively). Age >44years was an independent negative prognostic factor for DSS (*P* = 0.037) (Table 2).

### Acute toxicities

No grade 5 acute toxicity events (i.e. death) were observed in the matched sample during treatment. The rate of severe acute toxicities (grade 3–4) was higher in NACT + CCRT group relative to the CCRT group, however this difference was not statistically significant. A total of 110 patients (43.3%, 110/254) in the NACT + CCRT group and 91 (35.8%, 91/254) in the CCRT group experienced grade 3–4 acute treatment related adverse events (*P* = 0.102). The most common grade 3–4 hematological toxicity was leucopenia in 19.7% patients (*n* = 50) in the NACT + CCRT group and 13.4% patients (*n* = 34) in the CCRT group (*P* = 0.073). Grade 3–4 thrombocytopenia was found in 13 patients (5.1%, 13/254) and 7 patients (2.8%,7/254) in the NACT + CCRT group and CCRT group, respectively (*P* = 0.254). The most frequently recorded grade 3–4 non-hematological acute treatment related adverse events were mucositis, with 28.3% patients (*n* = 72) in the NACT + CCRT group and 20.9% patients (*n* = 53) in the CCRT group (*P* = 0.064). (Table 3)

## Discussion

To the best of our knowledge, this is the first study to explore the use of NACT followed by CCRT in the treatment of NPC patients diagnosed with CNN. We demonstrated that NACT followed by CCRT could improve DSS, DFS and DMFS in this particular group of patients, when compared with CCRT alone. After adjustment with other clinical factors in multivariate analysis, NACT + CCRT remained associated with a significant reduction in the rate of death, progress and distant metastasis. These results might give us some directions to select proper patients who might benefit most from the combination of NACT and CCRT, and help guide us to use NACT more effectively.

With the extensive adoption of CCRT as well as the emergence of highly precise RT techniques such as IMRT, the local-regional control rate in NPC has been considerably improved, with distant metastasis taking over as the predominant mode of treatment failure^16^,^17^,^18^,^19^. Therefore, NACT gradually came to our attention, due to its potential of reduction in both local and distant failures, although no significant improvement in overall survival attributable to NACT has previously been observed^20^,^21^. Our study demonstrated that NACT could significantly improve the survival of the specific group of patients with CNN. It was consistent with several studies which showed that NACT could significantly reduce the hazard of progression and distant failure in locally advanced NPC followed by CCRT^8^,^12^,^13^,^22^. Song *et al*^12^. reported that NACT + CCRT performed significant treatment effect in progressive free survival (HR = 0.66, 95% CI 0.49–0.90) and distant metastasis failure-free survival (HR = 0.60, 95% CI 0.39–0.98), when comparing with CCRT alone.

However, other studies failed to show any survival benefit from adding NACT to CCRT^14^,^15^. Because of the discordance of results received in different retrospective and clinical trials, the role of adding NACT to CCRT remains controversial^8^,^9^,^10^,^11^. The 2015 NCCN Guidelines^®^ only recommends it as a category 3 option for patients with NPC with T1,N1-3 or T2-4, any N lesions^5^. One possible explanation for the failure of NACT to improve survival in previous studies was that NACT might only be of benefit in certain high-risk group of patients with high potential for metastasis, but not all general advanced stage III-IV candidates enrolled in clinical trials. The predominant advantage of NACT was that it might be helpful eradicating the distant micro-metastases existing before radiation, not the new distant metastases emerged after treatment^23^. So the patients who might benefit most from NACT would be those who most likely had distant micro-metastases before radiation, such as patients with CNN in our study, but not all general stage III-IV patients^9^. The incidence of CNN in NPC patients with positive regional node metastases was as high as 44%. For this large number of patients who experienced poor DMFS^4^, appropriate treatment should be warranted.

Subgroup analyses showed that cisplatin-based NACT were generally tolerable, and no difference in survival was observed between different regimens, which were consistent with previous studies^24^,^25^. Over 90% of patients (*n* = 229) received two or more cycles of NACT, and no significant difference in the treatment efficacy between different cycles of chemotherapy was reported, with the exception of an improvement in RRFS using NACT ≤2 cycles. However, the small number of patients receiving >2 cycles of NACT (*n* = 51) suggests that these findings would require confirmation with larger studies.

Similar to other studies, acute toxicities were acceptable^8^,^9^. Although the rates of severe acute adverse events were higher in NACT + CCRT group, particularly with regards to leucopenia and mucositis, most of these toxicities were reversible.

A limitation of this study is the retrospective nature and single institute design. Although the propensity score match was used to eliminate personal bias between two groups, the results from larger, prospective randomized trials are required. Moreover, with the relatively short follow-up time (median, 50 months) and lack of complete late toxicity data, a long-term investigation was necessary.

In conclusion, NACT followed by CCRT could achieve better survival compared with CCRT alone in NPC patients diagnosed with CNN by reducing the risk of death, tumor progression and distant metastasis. Randomized trials are needed to further validate the value of NACT in this specific group of patients.

## Methods

### Patients

A total of 792 consecutive patients in previous study with newly histological-proven T1-4N1-3M0 NPC diagnosed with CNN based on MRI between January 2007 and December 2009 were enrolled in this study^4^. All patients were treated by definitive RT ± chemotherapy according to our institutional protocol. Restaging was performed according to the American Joint Committee on Cancer (AJCC) 2010 staging system^26^. The study was approved by the Research Ethics Committee of Sun Yat-sen University Cancer Center. All the methods were carried out in accordance with approved guidelines of our institute. Written informed consent was obtained from all patients prior to therapy.

Inclusion criteria included: (1) consecutive patients between January 2007 and December 2009 with a pathological diagnosis of nasopharyngeal squamous cell carcinoma; (2) complete MR images of the nasopharynx and neck; (3) all patients were diagnosed with cervical nodal necrosis based on MRI; (4)all patients were treated with concurrent chemoradiotherapy. Exclusion criteria included: 1) patients without complete MR images of the nasopharynx and neck; (2) patients without cervical nodal metastasis; (3) patients with at least one distant metastasis; (4) patients who did not have concurrent chemotherapy or had not completed radiation therapy. A total of 209 patients who did not receive CCRT were excluded from our study (147 patients received NACT + RT, 62 patients received RT alone). Of the 583 patients treated with CCRT, 262 were treated with NACT followed by CCRT while the remaining 321 patients received CCRT alone. The baseline characteristics of the patients were not balanced in two groups prior to matching. Patients in the NACT + CCRT group had more advanced T stage (T4), N stage (N3) and clinical stage (stage IV) disease relative to the unmatched CCRT arm (all P < 0.05). Demographic and clinical variables used to derive the propensity score model included age, gender, histological type, T stage, N stage, clinical stage and RT technique. Eventually, 508 patients were matched on the propensity score to create two groups each containing 254 patients. The characteristics of the patients were well-balanced between the propensity-matched groups (Table 4).

### Work-up

**Figure 2:**
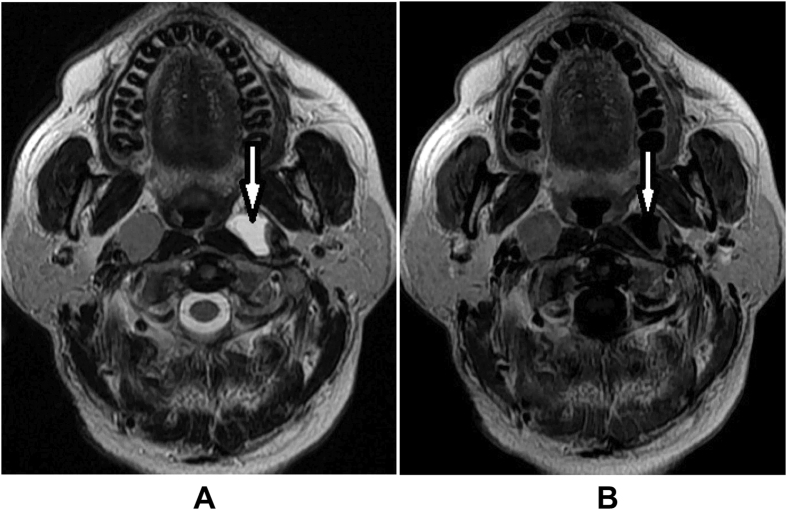
Appearance of a necrotic lymph node on MR images in a patient with NPC. (**A**) Axial T2-weighted (3300–5500/81–114) and (**B**) contrast-enhanced T1-weighted (400–600/9–16) MR images in a 45-year-old man show a left retropharyngeal lymph node with necrosis (arrows).

Pretreatment evaluation consisted of a complete physical examination, hematologic and biochemistry profiles, MRI scans of the nasopharynx and neck, chest radiography or computed tomography (CT), abdominal sonography, and emission computed tomography (ECT) or whole-body positron emission tomography/CT. Nasopharynx and cervical MR images were available for all patients and were used to assess lymph nodes status. The imaging protocol and the criteria for diagnosis of CNN have been reported in detail in previous study^4^. The criteria for diagnosis of CNN on MRI were a focal area of high signal intensity on T2WI images or a focal area of low signal intensity on T1WI + C images with or without a surrounding rim of enhancement. (Fig. 2).

### Treatment

All patients were treated with definitive RT covering the nasopharynx and retropharyngeal lymph nodes within the primary target. Whole-neck irradiation was performed in all cases. Over half of the patients (264/508; 52.0%) were treated by conventional techniques, 45.7% (232/508) by intensity modulated radiation therapy (IMRT), and 2.3% (12/508) by three-dimensional conformal radiation therapy (3D-CRT). The details of the RT techniques used at our institute have been reported previously^16^,^27^.

The NACT regimens included PF (75–80 mg/m^2^ cisplatin on day 1 and 800 mg/m^2^/d fluorouracil civ on days 1–5), TPF (70–75 mg/m^2^ docetaxel on day 1, 70–75 mg/m2 cisplatin on day 1 and 650–700 mg/m2/d fluorouracil civ on days 1–5) or TP (75 mg/m^2^ docetaxel on day 1 and 75 mg/m^2^ cisplatin on day 1); the regimens were repeated every 3 weeks. PF, TPF, TP were administered in 132, 53 and 69 patients, respectively. Two-hundred and three patients received 2 cycles NACT and only 51 patients received over 3 cycles. The CCRT regimen was 80–100 mg/m2 cisplatin repeated every 3 weeks or 40 mg/m^2^ weekly. Patients receiving other chemotherapy regimens were excluded from this study.

### Follow-up

Follow-up was calculated from the day of diagnosis to the date of the event or last follow-up visit. All patients were followed-up every 3 months for the first 3 years, every 6 months for the next 2 years, and then annually. Toxicity was assessed according to the Radiation Therapy Oncology Group criteria^28^ or the Common Terminology Criteria for Adverse Events [CTCAE] v3.0^29^. Disease-specific survival (DSS) was calculated from the day of diagnosis to death caused by cancer at the date of last follow-up; disease–free survival (DFS) was calculated from the day of diagnosis to loco-regional relapse, distant relapse or tumor-related death; regional recurrence–free survival (RRFS) was calculated from the day of diagnosis to regional relapse; distant metastasis–free survival (DMFS) was calculated from the day of diagnosis to first observation of distant lesion(s).

### Statistical analysis

A propensity score matching method^30^ was performed to match the patients from the NACT + CCRT group to comparable patients in the CCRT group on a 1:1 basis. Propensity scores were calculated by logistic regression for patients based on the listing covariates: age, gender, histological type, T stage, N stage, clinical stage and RT techniques. Covariates balance between groups was examined by chi–square test or fisher exact test (categorical variable) as appropriate. Survival rates were calculated using the Kaplan-Meier method and differences were compared by using the log-rank test. A multivariate Cox proportional hazards model was used to calculate hazard ratios (HR), 95% confidence intervals (CI) and test the independent significance of different factors by backward elimination of insignificant factors. A two-tailed P-value less than 0.05 was considered statistically significant. All statistical analyses were performed using SPSS v 22.0 (Chicago, IL, USA) and STATA version14.0.

## Additional Information

**How to cite this article**: Lan, M. *et al*. Neoadjuvant chemotherapy followed by concurrent chemoradiotherapy versus concurrent chemoradiotherapy alone in nasopharyngeal carcinoma patients with cervical nodal necrosis. *Sci. Rep.* **7**, 42624; doi: 10.1038/srep42624 (2017).

**Publisher's note:** Springer Nature remains neutral with regard to jurisdictional claims in published maps and institutional affiliations.

## Supplementary Material

Supplementary Information

## Figures and Tables

**Table 1:** Patterns of treatment failure in the propensity-matched cohort of 508 patients.

Treatment failure pattern	NACT + CCRT (*n*)	CCRT (*n*)	*P*-value†	Total
Distant metastases alone			0.001	
Bone	7 (5.9)	17 (14.5)		24
Lung	12 (10.2)	16 (13.5)		28
Liver	12 (10.2)	16 (13.5)		28
Bone + Lung	2 (1.7)	6 (5.1)		8
Bone + Liver	4 (3.4)	4 (3.4)		8
Lung + Liver	1 (0.8)	4 (3.4)		5
Bone + Lung + Liver	4 (3.4)	8 (6.8)		12
Brain	0 (0.0)	2 (1.7)		2
Other*	1 (0.8)	2 (1.7)		3
Total	43 (36.4)	75 (63.6)		118
Distant metastasis + recurrence	6 (66.7)	3 (33.3)		9
Local-regional recurrence alone			0.530	
Local	21 (55.3)	17 (44.7)		38
Regional	10 (41.7)	14 (58.3)		24
Local + regional	3 (25.0)	9 (75.0)		12
Total	71 (38.8)	112 (61.2)	<0.001	183

NACT = neoadjuvant chemotherapy, CCRT = concurrent chemoradiotherapy. ^*^Mediastinal, para-aortic lymph node metastases or metastases to other organs. ^†^P-values were calculated using the χ^2^ test (or Fisher’s exact test, if the expected number was less than five in at least 25% of the cells). Numbers in parentheses are percentages.

**Table 2:** Multivariate analysis of variables correlated with various clinical endpoints in the propensity-matched cohort of 508 patients.

Characteristic	5y-DSS		5y-DFS		5y-RRFS		5y-DMFS	
	HR(95% CI)†	*P* value*	HR(95%CI)†	*P* value*	HR(95%CI)†	*P* value*	HR(95%CI)†	*P* value*
Gender(male)	1.94 (1.17–3.22)	0.010	1.49 (1.04–2.12)	0.028	1.21 (0.57–2.58)	0.619	1.91 (1.18–3.09)	0.008
Age(>44years)	1.49 (1.02–2.17)	0.037	0.91 (0.68–1.22)	0.527	0.70 (0.35–1.40)	0.308	1.02 (0.71–1.47)	0.919
T stage	1.29 (0.98–1.70)	0.067	1.29 (1.06–1.57)	0.013	0.98 (0.79–1.24)	0.282	1.46 (1.14–1.88)	0.003
Clinical stage	1.39 (0.94–2.06)	0.098	0.92 (0.69–1.22)	0.549	0.94 (0.72–1.30)	0.365	1.35 (0.93–1.97)	0.117
Treatment (NACT + CCRT)	0.60 (0.41–0.88)	0.009	0.54 (0.40–0.73)	<0.001	0.57 (0.29–1.12)	0.102	0.49 (0.34–0.71)	<0.001

NACT = neoadjuvant chemotherapy, CCRT = concurrent chemoradiotherapy, DSS = disease-specific survival, DFS = disease-free survival, RRFS = regional recurrence-free survival, DMFS = distant metastasis-free survival. ^†^Hazard ratios and ^*^P values were calculated using the Cox proportional hazards model.

**Table 3:** Severe acute toxicities (grade 3–4) in the propensity-matched cohort of 508 patients.

Variable	NACT + CCRT group (*n* = 254)	CCRT group (*n* = 254)	*P* value*
Hematologic			
Leukopenia	50 (19.7)	34 (13.4)	0.073
Neutropenia	43 (16.9)	30 (11.8)	0.129
Anemia	6 (2.36)	1 (0.39)	0.122
Thrombocytopenia	13 (5.12)	7 (2.76)	0.254
Non-hematologic			
Mucositis	72 (28.3)	53 (20.9)	0.064
Dysphagia	55 (21.7)	50 (19.7)	0.661
Nausea/vomiting	48 (18.9)	32 (12.6)	0.067
Dermatitis	24 (9.45)	29 (11.4)	0.562
Xerostomia	12 (4.72)	20 (7.87)	0.201
Hepatoxicity	5 (1.97)	0 (0.00)	0.061
Nephrotoxicity	0 (0.00)	0 (0.00)	—
Neurotoxicity	0 (0.00)	0 (0.00)	—

NACT = neoadjuvant chemotherapy, CCRT = concurrent chemoradiotherapy. Numbers in parentheses are percentages. ^*^P-values were calculated using the Chi-square test (or Fisher’s exact test, if the expected number was less than five in at least 25% of the cells).

**Table 4:** Baseline characteristics of NPC patients diagnosed with CNN and treated with CCRT.

Characteristic	The original unmatched cohort			The propensity-matched cohort		
	NACT + CCRT (*n* = 262)	CCRT (*n* = 321)	*P* value	NACT + CCRT (*n* = 254)	CCRT (*n* = 254)	*P* value
Age (years)			0.025			0.533
≤4	149 (56.9)	152 (47.4)		143 (56.3)	135 (53.1)	
>44	113 (43.1)	169 (52.6)		111 (43.7)	119 (46.9)	
Gender			0.635			1.000
Male	197 (75.2)	235 (73.2)		191 (75.2)	190 (74.8)	
Female	65 (24.8)	86 (26.8)		63 (24.8)	64 (25.2)	
Histological type			0.590			1.000
WHO III + II	260 (99.2)	320 (99.7)		252 (99.2)	253 (99.6)	
WHO I	2 (0.8)	1 (0.3)		2 (0.8)	1 (0.4)	
T stage			0.001			0.337
T1	5 (1.9)	12 (3.7)		5 (2.0)	8 (3.1)	
T2	48 (18.3)	73 (22.7)		48 (18.9)	52 (20.5)	
T3	125 (47.7)	177 (55.2)		125 (49.2)	135 (53.1)	
T4	84 (32.1)	59 (18.4)		76 (29.9)	59 (23.2)	
N stage			<0.001			0.306
N1	72 (27.5)	135 (42.1)		72 (28.3)	84 (33.1)	
N2	79 (30.2)	99 (30.8)		79 (31.1)	83 (32.7)	
N3	111 (42.3)	87 (27.1)		103 (40.6)	87 (34.2)	
Clinical stage			<0.001			0.051
II	10 (3.8)	33 (10.3)		10 (3.9)	13 (5.1)	
III	80 (30.5)	151 (47.0)		80 (31.5)	104 (40.9)	
IV	172 (65.7)	137 (42.7)		164 (64.6)	137 (54.0)	
RT technique			0.022			0.075
IMRT	109 (41.6)	164 (51.1)		106 (41.7)	126 (49.6)	
2DRT/3DCRT	153 (58.4)	157 (48.9)		148 (58.3)	128 (50.4)	

NPC = nasopharyngeal carcinoma, CNN = cervical nodal necrosis, NACT = neoadjuvant chemotherapy, CCRT = concurrent chemoradiotherapy, RT = radiation therapy, IMRT = intensity modulated radiation therapy, 2DRT = conventional radiation therapy, 3D-CRT = three-dimensional conformal radiation therapy. P values were calculated using the χ^2^ test (or Fisher’s exact test, if the expected number was less than five in at least 25% of the cells).
