# Supplementary material for: Neoadjuvant chemotherapy followed by concurrent chemoradiotherapy versus concurrent chemoradiotherapy alone in nasopharyngeal carcinoma patients with cervical nodal necrosis
Source: Sci Rep. 2017 Feb 17;7:42624. doi: 10.1038/srep42624 (PMC5314371; doi:10.1038/srep42624)

Title: Neoadjuvant chemotherapy followed by concurrent chemoradiotherapy versus concurrent chemoradiotherapy alone in nasopharyngeal carcinoma patients with cervical nodal necrosis

Author list: Mei Lan, M.D. †1, Chunyan Chen, M.D. †1, Ying Huang, M.D. 1 , Li Tian, M.D. 2, Zhijun Duan, M.D. 3, Fei Han, M.D. 1, Junfang Liao M.D. 1, Meiling Deng, M.D. 1, Terence T. Sio, M.D., M.S. 4, Anussara Prayongrat, M.D.5, Lie Zheng, M.D. 2, Shaoxiong Wu, M.D. *1 , Taixiang Lu, M.D* 1.

Supplementary Table. Univariate predictors of disease-specific survival , disease-free survival, regional recurrence-free survival and distant metastasis-free survival in the propensity-matched cohort of 508 patients.

Characteristic	*N* (%)	5y-DSS	*P* value*	5y-DFS	*P* value*	5y-RRFS	*P* value*	5y-DMFS	*P* value*
**Gender**			0.007		0.029		0.7		0.009
Male	381(75.0)	73.8		59.1		91.4		71.8	
Female	127(25.0)	87.7		71.4		93.8		82.9	
**Age(years)**			0.01		0.863		0.242		0.444
≤44	278(54.7)	81.7		61.4		90.3		75.6	
＞44	230(45.3)	71.9		63.6		94.4		73.3	
**T stage**			0.037		0.121		0.352		0.066
T1	13(2.5)	82.1		84.6		92.3		91.7	
T2	100(19.7)	82.9		66.7		89.5		83.5	
T3	260(51.2)	79.1		63.5		94.2		73.8	
T4	135(26.6)	69.1		54		94.7		67.2	
**N stage**			0.181		0.658		0.565		0.521
N1	156(30.7)	81.8		59.2		89.7		77.3	
N2	162(31.9)	73.7		60.2		92.7		72.1	
N3	190(37.4)	77.1		66		93.5		74.3	
**Clinical stage**			0.049		0.129		0.547		0.075
II	23(4.5)	86.7		54.2		88.9		85.9	
III	184(36.2)	82.5		66		91.5		78.3	
IV	301(59.3)	73.5		61.1		94.6		71.6	
**Treatment**			0.022		<0.001		0.053		<0.001
NACT+CCRT	254(50.0)	82.1		70.3		94.3		81.9	
CCRT alone	254(50.0)	72.5		54.1		89.6		67.3	
**RT technique**			0.244		0.666		0.442		0.22
IMRT	232(45.7)	74.9		60.6		90.6		71.9	
2DRT/3DCRT	276(54.3)	79.6		63.7		93.3		77	

Abbreviations: NACT= neoadjuvant chemotherapy, CCRT= concurrent chemoradiotherapy, RT= radiation therapy, IMRT= intensity modulated radiation therapy, 2DRT= conventional radiation therapy, 3D-CRT= three-dimensional conformal radiation therapy, DSS= disease-specific survival, DFS= disease-free survival, RRFS= regional recurrence-free survival, DMFS=distant metastasis-free survival. * P values were calculated using the log-rank test. Numbers in parentheses are percentages.

Supplementary Figure 1. Kaplan–Meier disease-specific survival (A), disease-free survival (B), regional recurrence-free survival (C) and distant metastasis-free survival (D) curves for patients with different NACT regimens.

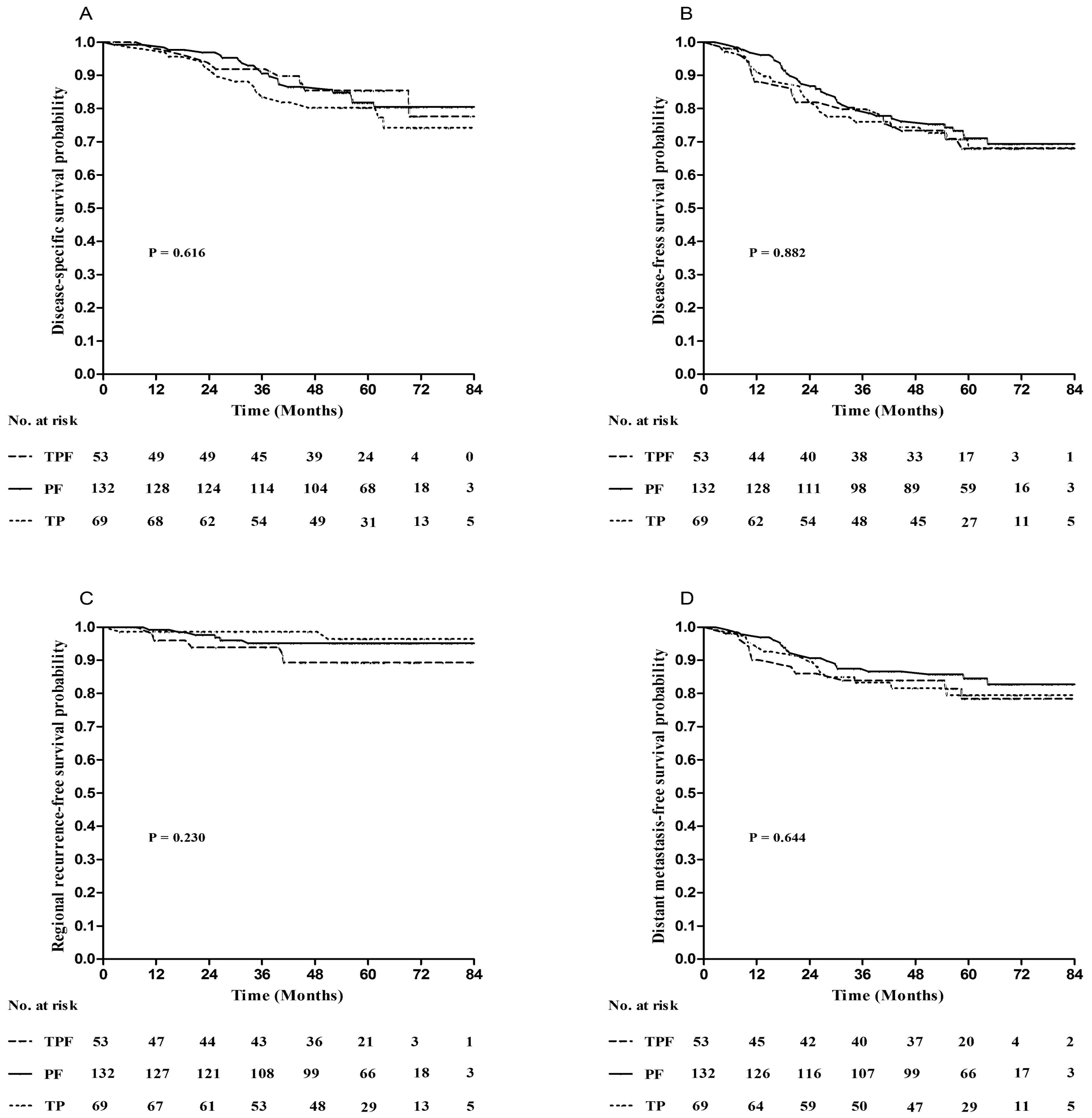

Supplementary Figure 2. Kaplan–Meier disease-specific survival (A), disease-free survival (B), regional recurrence-free survival (C) and distant metastasis-free survival (D) curves for patients with different cycles of NACT.

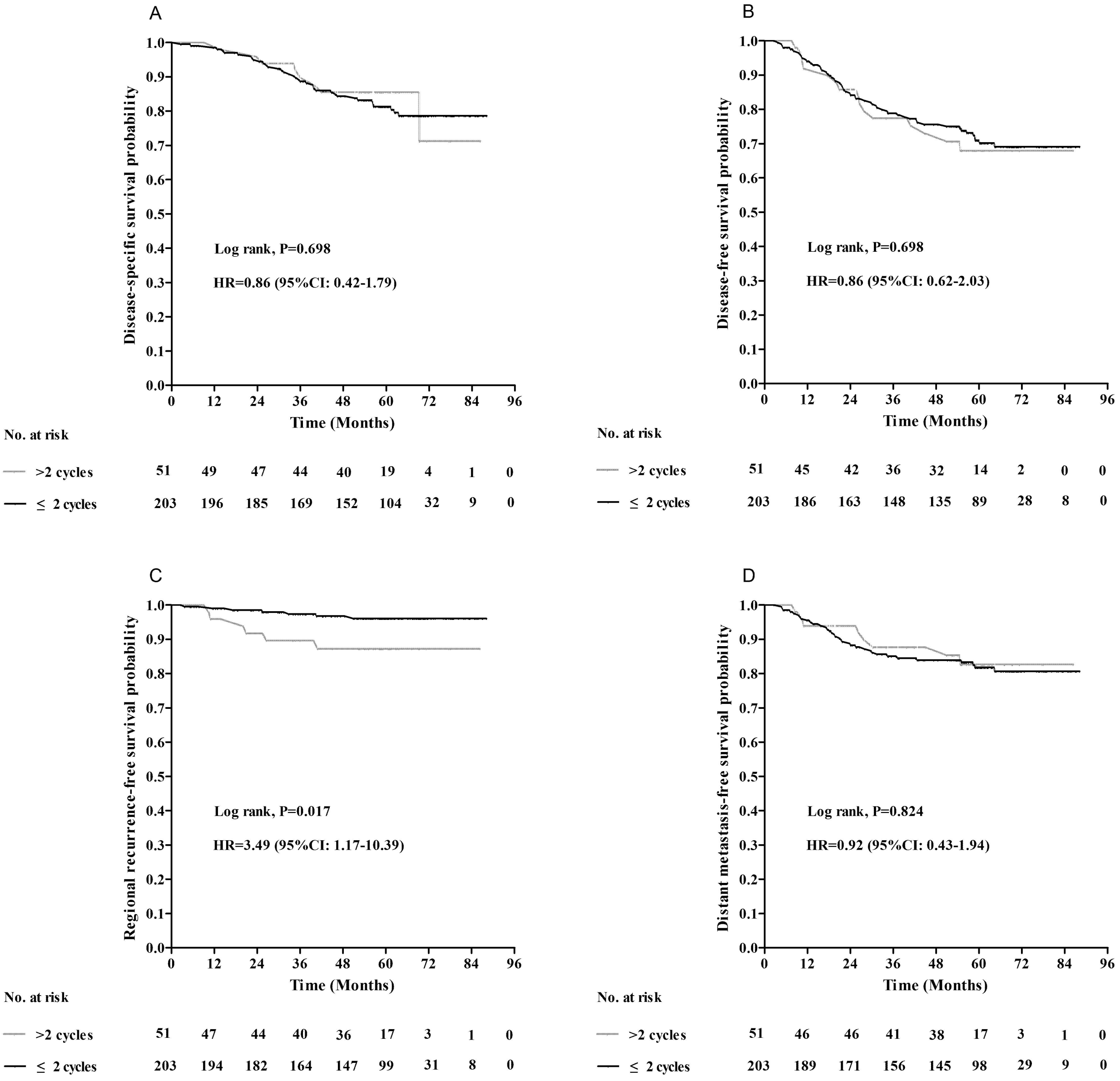

Supplement: Supplementary Information [file srep42624-s1.pdf]
